# Cure State Sensing of Polymethylmethacrylate Using a Vibrating Axial Probe

**DOI:** 10.3390/s24134365

**Published:** 2024-07-05

**Authors:** Avonley Nguyen, Quang V. Nguyen, Daniel Funk

**Affiliations:** 1 RFA Systems, LLC., Aldie, VA 20105, USA; avonley@rfasystems.com; 2 WaveTest, LLC., Cincinnati, OH 45208, USA; dfunk.cinci@gmail.com

**Keywords:** acoustic resonance spectroscopy, rheological properties sensing, bone cement

## Abstract

A new axially vibrating sensor based on an audio voice coil transducer and a lead zirconate titanate (PZT) piezoelectric disc microphone was developed as a probe for the measurement of in vitro rheological fluid properties, including curing progress for polymethylmethacrylate (PMMA) mixtures with important uses as bone cement in the field of orthopedics. The measurement of the vibrating axial sensor’s acoustic spectra in PMMA undergoing curing can be described by a damped harmonic oscillator formalism and resonant frequency (ca. 180 Hz) shift can be used as an indicator of curing progress, with shifts to the blue by as much as 14 Hz. The resonant frequency peak was measured in 19 different 4.0 g PMMA samples to have a rate of shift of 0.0462 ± 0.00624 Hz·s^−1^ over a period of 400 s while the PMMA was in a dough state and before the PMMA transitioned to a hard-setting phase. This transition is unambiguously indicated by this sensor technology through the generation of a distinct circa 5 kHz high-Q under-damped ring-down response.

## 1. Introduction

In 1941, polymethylmethacrylate (PMMA) was first used in medicine as a dental prosthetic, and then subsequently as a substitute for bone in cranial surgery. It was in the late 1950s when its use widened into orthopedics for the fixation of total joint implants. Since that time, PMMA has become widely used and is the gold standard for the fixation of orthopedic implants in surgery [1]. These and other novel applications of PMMA in medicine are described in a review article by Webb and Spencer [2]. Due to PMMA’s properties of being soft and pliable, it is used as grout to fill small interstitial spaces in the bone and implant to provide mechanical fixation. For that reason, knowing the viscosity of PMMA during surgery is important; if it is too thin, it will flow out of the interstices and if it is too thick, the PMMA will not enter the interstices. In either case, the result is the poor fixation of the implant.

The mechanical properties of PMMA have been extensively studied in recent years, for example, to look at the effects of temperature on high rate deformation [3], the effects of compression versus tension [4], the effects of contaminants [5], or the effect on the strength of PMMA resulting from the addition of antibacterial drugs [6]. Additionally, manufacturers of medical-grade PMMA provide various formulations that have different viscosities and cure times to allow the surgeon to select the most effective properties for their particular use case. The variations in environmental temperature and humidity, the presence of blood or tissues, and water can all change the cure time for PMMA during a surgical procedure [7]. The typical amount of time that a practitioner has to work quickly with the PMMA before it is too viscous can vary widely depending on the formulation, manufacturer, and additives. The working time can range from 1 min to 10 min depending on the local conditions [1]. Once PMMA enters the ‘dough’ phase there is about a 5 min window of time for it to be applied as grout. After the dough phase, it suddenly transitions to the ‘setting’ phase where it rapidly cures while releasing a tremendous amount of heat due to the exothermic reaction between the monomer and polymer. This final stage can take anywhere between 30 s and several minutes to cure to a hardened state [1]. This wide range and variations in working times necessitate the need for a sensor that can detect the cure state of PMMA in a quantitative manner to provide the practitioner with an accurate assessment of the time remaining to complete the procedure or to mix a new batch of PMMA.

The current standard of care is for the practitioner to palpate a small sample of the PMMA in their hand to determine the firmness or stickiness as a gauge of when the cement is of the right viscosity for use [1]. This manner of testing is inherently inaccurate since the outside environment is significantly different from the in vivo cement environment. Even the warmth of the hand checking the cement changes its cure characteristics. At this time, there is no method or instrument able to determine the viscosity of the PMMA during use in the operating room. Although studies have shown that there is an optimal viscosity to use the PMMA, the present state of the art amounts to a guess based on the experience of the surgeon.

In situ methods to sense or measure the viscosity of fluids include acoustic or ultrasonic resonance spectroscopy (ARS), which was developed initially by Sinha and Olinger [8] to non-invasively monitor the contents inside munition canisters to identify whether a shell contains explosives or biological/chemical warfare substances. ARS utilizes an acoustic transmitter and receiver placed in contact with the outer walls of the vessel under investigation. By applying an acoustic excitation to the transmitter using an audio speaker, and then monitoring the resulting acoustic signal generated by the vessel using a microphone, the internal contents of the vessel can be determined by performing an FFT on the microphone signal to see the frequency components [8]. Furthermore, the container can be rotated, and the multiple ARS FFT spectra are collected and de-convoluted using a method similar to tomographic reconstruction to see the shape of the objects of varying densities inside the container under test [8]. This method could potentially work to monitor the spectra of PMMA as it cures through the changes in the speed of sound and the level of attenuation through the storage chamber. The ARS technique was later expanded to rapidly monitor and differentiate the constituents in drugs such as tablets and pills [9], and then, later, ARS was used to measure the bulk modulus of substances using differential ARS (DARS) [10].

All these methods require monitoring the acoustic signature of the PMMA inside a small chamber, which is not convenient or practical in the operating room. Lionetto and Maffezzoli [11] demonstrated the measurement of the cure state of thermosetting resins using an ultrasonic time-of-flight propagation sensor where the resin sample was placed in a chamber formed by two opposing ultrasonic transducers. In this work, the change in the speed of sound propagation and its attenuation through the material under testing allowed the determination of the complex longitudinal [11]. However, the physical arrangement of this type of sensor does not lend itself readily to measuring a quantity of PMMA awaiting to be dispensed. A similar concept to that used by [11], but with only a single ultrasonic transducer on one end of a cylindrical chamber containing epoxy resin to be monitored, was demonstrated by [12], where the authors showed that the reflected propagation of an ultrasonic sound wave through the sample can be used to determine the time of flight, and hence the speed of sound, through the sample. This was then related to a model of wave propagation through the material using a fitting parameter to the Weibull model of resin curing [12]. However, the same technique, also known as the ‘pulse echo’ ultrasonic testing method was applied to cured polymers including PMMA, low-density polyethylene (LDPE), and polyamide (PA); this resulted in cases of large errors of the modulus of elasticity of up to 98% when compared to the traditional destructive testing of the materials [13]. In this paper, the authors showed that using a linear model to correlate the pulse echo data with destructive tensile testing can reduce the error down to the 5% level [13].

Moving onto electromechanical resonators as sensors, we see a wide variety of techniques and many of these are summarized in a review article by Voglhuber-Brunnmaier and Jakoby [14]. These techniques can be broadly categorized into either electrodynamic resonators or electrical resonators, where the latter does not employ any mechanical motion [14]. In the area of electrodynamic resonators, sensor technologies include vibrating cantilevers, discs, spheres, bridges, tuning forks, surface acoustic wave (SAW) devices, U-shaped beams, helical coils, and microscopic wires in crossflow [14]. What these techniques all have in common is the use of a damped harmonic oscillator model (DHO) to describe the equations of motion. As such, the solution to the DHO can be characterized by a resonant frequency ω_0_ and the Q of the resonance, along with the amplitude and phase [14].

Significant research has been performed to model the physics of the microcantilever beam; its physical properties showed that by measuring and characterizing the amplitude and phase of a microcantilever vibrating in the fluid of interest, the density and viscosity of the fluid can be extracted using an elegant model of the cantilever–fluid interaction [15,16]. However, the authors of [17] suggest that microscale resonators may not be the best choice for the measurement of the rheological properties of non-Newtonian fluids, or fluids where there are strong dissipation effects that deteriorate the quality factor, or Q of the oscillator and, therefore, a larger macro-scale resonator consisting of a vibrating sphere on the end of a cantilever can be more accurate for the simultaneous measurement of fluid density and viscosity [17]. A study of larger scale tunable resonators using electromagnetically excited vibrating plates and vibrating wires were used to measure the viscosity of various liquids with good success [18].

The use of a hollow electromagnetically excited tube to produce both torsional and bending modes simultaneously to measure both the fluid density and viscosity with good sensitivity was described in [19]. The authors of [20] showed that microcantilevers could indeed be used for fluids with complex rheological properties by extracting the complex shear modulus from the microcantilever’s deflection spectrum [20]. However, the microcantilever used here has electrical paths embedded in it to provide electromagnetic excitation and suffers from dominant electrical coupling to the fluid, which this limits its use in non-electrically conductive liquids [21]. Resonant MEMS-based microcantilevers, however, work very well for characterizing non-electrically conductive fluids such as seven different alkanes, as demonstrated by [22]. Finally, vibrating microcantilever beams were used to measure fluid density and viscosity using a novel flexural wave analysis technique using a laser Doppler vibrometer to measure the standing wave profile, which was correlated to the fluid properties through a matrix fitting to the equations of motion [23]. More recently, a study on waterproofing aluminum nitride (AlN) piezoelectric MEMS viscosity meters showed promise in allowing the use of MEMS sensors in electrically conductive fluids, but this still does not provide the necessary sensor technology for measuring highly viscous dough-like states of PMMA [24].

A review of these methods indicates that although they all show promise in determining PMMA viscosity; none of them are suitable for intraoperative use due to their complexity, or incompatibility with the high viscosity of PMMA, or they are too physically large to be used as tool in the operating room environment. What is needed is a new approach to determining viscosity that is compact, easy to use, and has the qualities necessary to be used in the surgical suite. The sensor described in this paper has the pre-requisites to be made into a compact and easy to use hand-held surgical tool for a surgical practitioner in the operating room. In this paper, we will show a simple yet novel sensor utilizing an axially vibrating probe excited by a small electromagnetic transducer that can be fitted to either a hand-held probe instrument or perhaps inside a vessel used for mixing or dispensing the PMMA cement. The novelty of this new technology is that it will permit, for the first time, an interoperative instrument for use by an orthopedic surgeon to measure, in vivo or in vitro, the relative viscosity of a mixture of PMMA that has a ticking expiration clock measured in minutes. The use of this type of sensor in the operating room will allow a surgeon to determine whether or not the batch of recently prepared PMMA is still good to be used as surgical grout for bone implants; furthermore, it may be used to tell if the PMMA grout already applied to the bone has reached its final cure state. This is important since PMMA that has been allowed to harden too long will not adhere to bone properly and can cause major surgical reworking. This is both costly and dangerous for the patient and should be avoided or prevented, if possible. This technology now provides a sure way of knowing the PMMA cure state without ambiguity.

This paper is organized as follows: in Section 2, we discuss the sensor technology, how it works (including the theory of operation), and how it is built and tested; in Section 3, we describe the experimental results of the testing and discuss the significance of the findings; in Section 4, we discuss the experimental results and their interpretation; and in Section 5, we present a summary of the findings in the Conclusions.

## 2. Materials and Methods

From the above many techniques described for monitoring the rheological properties of fluids, the one employed by [19] seems the closest to the method that we independently developed and used for this paper. We wanted to use a large electromagnetic voice coil actuator and a simple macro-scale cylindrical probe in order to overcome the high levels of viscous damping with low-viscosity PMMA having viscosities in the 1000 Poise (10 Pa·s) range at a temperature of 20 °C [25], which would render the nano- and microcantilever-based methods ineffective due to the mismatch of the viscosity range of the PMMA versus the fluids compatible with microcantilever resonators like water, alkanes, and silicon oil, which have viscosities in the 0.01 Poise (10^−3^ Pa·s) range [17]. The system that we developed for sensing the viscosity and firmness of the dough state of PMMA is a simple audio speaker voice coil actuator capable of overcoming the high viscosity of PMMA.

**Figure 1:**
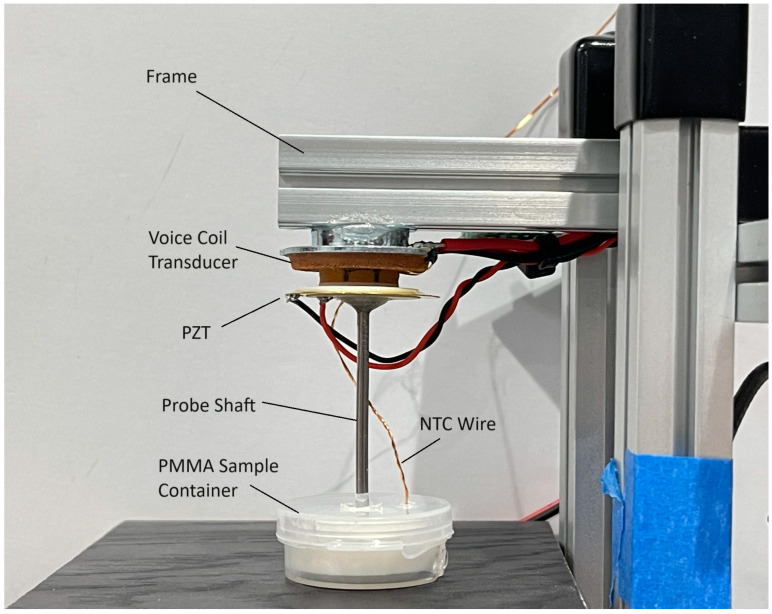
A closeup of the sensor system. For scale, the PMMA sample container is 25 mm in diameter (internal), the probe shaft is 2.3 mm in diameter, and the voice coil transducer is 27 mm in diameter.

Figure 1 shows a photograph of the experimental apparatus and sensor system that we developed for this paper and shows a speaker actuator consisting of a voice coil/magnet motor and associated suspension system but minus the speaker cone. This voice coil transducer is connected to a thin piezoelectric transducer disc, which is in turn mounted to a stainless-steel cylindrical probe tip. This arrangement allows for the force transmitted between the voice coil and the probe to be monitored by the voltage signal generated by the piezoelectric disc, which is sandwiched in between. The small probe tip can be simply inserted into the cement that is under test, either directly or through a small port in the chamber holding the cement paste. The components of the sensor probe are all cemented to each other using epoxy cement. As the voice coil transducer has a radial suspension designed for the 1-dimensional actuation of an audio speaker cone, we can assume that, for small probe motion displacements, the motion is 1-dimensional (purely axial). Thus, the probe tip moves axially along its axis, as opposed to cantilever beam oscillators which rely on a bending mode perpendicular to the beam axis. Since the mass of the sensor probe shaft is small relative to the mass of the voice coil actuator, the resonant frequency of the system is dominated by the resonant frequency of the voice coil actuator (with the overall system resonant frequency in the 180 Hz range). This lower frequency, combined with an axial motion dictated by the voice coil suspension, permits the use of a 1-dimensional damped harmonic oscillator (DHO) model to describe the vibration of this system. The assumption of a 1-dimensional damped harmonic oscillator model of a dynamic speaker voice coil transducer is further confirmed by the fact that dynamic loudspeaker transducers have been extensively studied and characterized using a 1-dimensional equivalent electrical circuit analogy for the accurate modeling of speaker systems, popularly known in the audio world as the ‘Thiele–Small’ model for dynamic speaker transducers [26,27].

**Figure 2:**
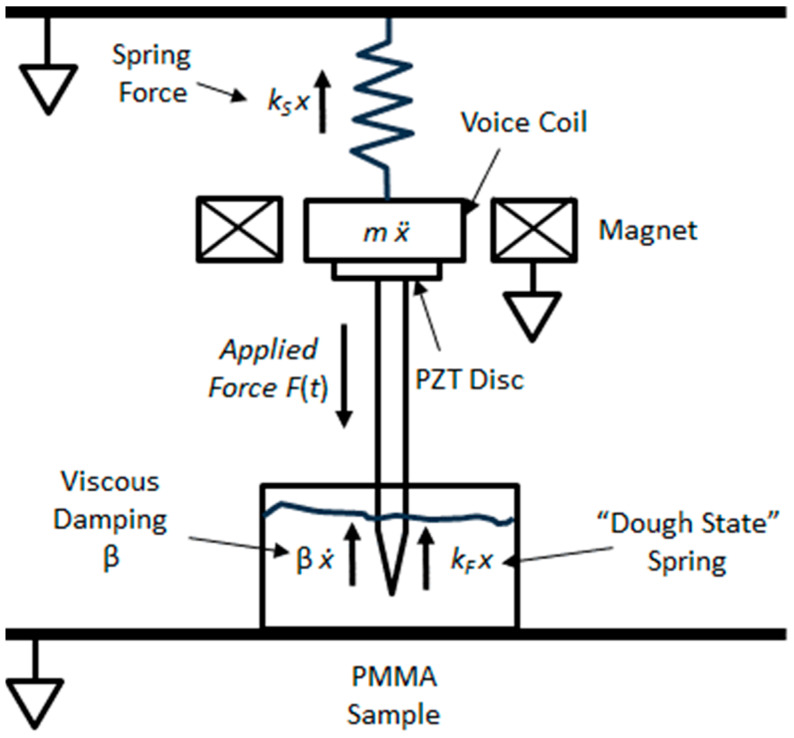
Schematic diagram of the forces on the 1-dimensional damped spring mass system. Note the additional term for the “dough state spring” constant resulting from the change in the compliance of the PMMA as it hardens.

Referring to the simplified mass–spring system representing the sensor, as shown Figure 2, we have a mass *m* connected to a frame (mechanical ground) via a suspension with spring constant *k_S_*, and, on the other side of the mass *m*, a thin piezoelectric transducer disc is mounted directly on the transducer using epoxy. The mass is electromagnetically coupled to a coaxial cylindrically wound voice coil which drives the mass with a time-dependent force given by *F*(*t*) = *A*_0_ cos(ω*t*), where *A*_0_ is the maximum amplitude of the motion along direction *x*, oscillating at a frequency ω. A small-diameter stainless-steel probe is affixed to the second face of the piezoelectric disc with epoxy and the distal end of the probe is inserted about 5 mm under the liquid surface of the PMMA under testing. Figure 2 shows a familiar classical force-driven, 1-dimensional harmonic oscillator with damping provided by the probe tip immersed in the liquid PMMA, not unlike a fluid based dashpot, which provides the viscous damping force *b dx*/*dt*, where *b* is the damping constant. There is one more aspect of this system that we need to model that is not obvious: as the PMMA cures, it hardens, and its elastic modulus increases while its loss modulus decreases. The increase in the elastic modulus is analogous to the probe tip pushing and pulling on a cure-state-dependent spring of constant *k_F_* (*φ*), where *φ* is the cure state progress variable, and the loss modulus is related to the damping constant *b*. Looking at Figure 2, if we write the force balance using the equation of motion along 1-dimension *x*, we obtain the following:

(1) $$m\frac{d^{2}x}{dt^{2}}=-k_{S}x-k_{F}(\varphi )x-b\frac{dx}{dt}+F(t)$$

The solution to this classic forced damped harmonic oscillator (DHO) is well known, and for example, has a solution of the following form [28]:

(2) A=F0mω02−ω22+(ω γ)2.

Here, *A* is the steady state amplitude, and the damping factor γ is given by the following:

(3) $$\gamma =\frac{b}{m}.$$

The system resonant frequency ω_0_ is given by the following:

(4) $$\omega_{0}=\sqrt{\frac{k_{TOT}}{m}},$$

where *k_TOT_* = *k* + *k_F_*(φ). Note that the resonant frequency is a function of the cure state progress variable φ. The phase δ between *F*(*t*) and position *x*(*t*) is given by the following:

(5) $$\tan\delta =\frac{\omega \;\gamma (\emptyset )}{{\omega_{0}}^{2}-\omega^{2}}$$

with the position of the probe tip movement *x*(*t*) given by the following:

(6) $$x(t)=A_{0}\cos\left(\omega t-\delta \right).$$

From Equation (4), we can see that the resonant frequency *ω*_0_ will increase as the cement cures because the PMMA will increase in stiffness, and hence the spring constant, *k*_F_, will increase, since the mass m is constant. Intuitively, we know that increased stiffness in the PMMA will also mean that there will be an increase in the damping factor gamma, which means that the width of the resonant frequency peak will broaden in frequency space. A similar analysis using the equations describing the impedance of a speaker voice coil, as described by [26], would also yield an additional reactive term that varies with the compliance of the PMMA, which is similar to the term, *C*_mes_, in the notation of [26] used to describe the electrical compliance of the loudspeaker voice coil in an equivalent lumped-element electrical network, which has the property of a capacitance *C*_mes_ that is proportional to the velocity of the system [26].

**Figure 3:**
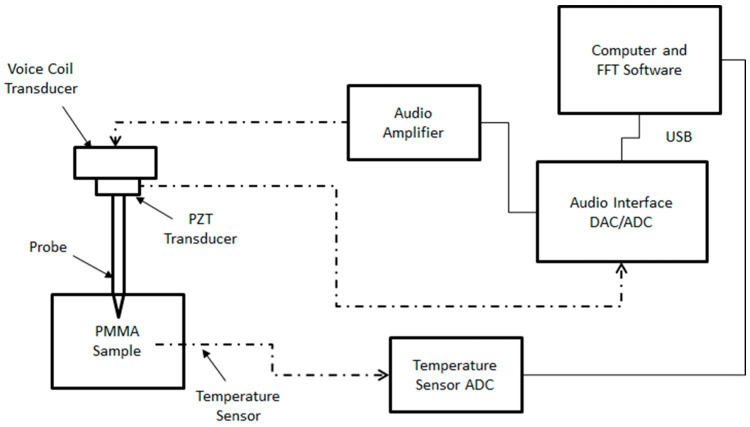
Simplified schematic diagram of the experimental setup. Note that the temperature sensor ADC was connected to the PC for power via USB.

The experimental setup in Figure 3 schematically shows an audio voice coil transducer (Aiyima, 27 mm dia., 4 ohms, 2 W) and piezoelectric transducer (generic 27 mm guitar pickup, 0.4 mm thick) as a microphone mounted to a cylindrical probe (stainless steel nail, 2.3 mm dia. × 40 mm long) with epoxy. The voice coil transducer top is mounted to a square cross-section ‘T-rail’ mounting system (MakerBeam XL), which provides the mechanical ground for the transducers and the PMMA sample container (24 mm dia. × 10 mm deep, HDPE). A PC computer equipped with an audio measurement software suite, which includes spectrum using FFT, and impulse response measurement software (REW version 5.20.4) controls a USB digital audio interface (Scarlett, Solo Gen 2) which provides the analog to digital (ADC) and digital to analog (DAC) that drives an audio amplifier (ST Electronics, TDA7297 with an 18 V linear power supply) and receives the high impedance voltage signal from the PZT transducer, respectively. The REW software provides a chirped sine wave sweep excitation signal to the power amplifier which then drives the audio voice coil from 4 Hz to 96 kHz while collecting the signal generated by the PZT. The raw time-series signal is captured at a 96 kHz sample rate and 24-bit resolution, and subsequently processed by the REW software to provide a spectrum in frequency space. The excitation amplitude from the amplifier to the voice coil was set at 1.00 Vpp (approximately 250 mW power delivered into a nominal 4-ohm voice coil) and measured using an oscilloscope (Owon, HDS24S). The voice coil transducer responds to the amplifier signal by effecting an axial motion onto the PZT transducer disc, which, in turn, passes that motion to the probe shaft. As all components are hard-mounted with epoxy cement in mechanical series, the exact force transmitted by the voice coil to the probe shaft is sensed by the piezoelectric transducer as a voltage signal that is linearly proportional to the force. The signal from the PZT was typically in the 2 V to 5 V range, which is well matched to the input stage of the ADC provided by the USB audio interface. By using a PZT mechanically in series with the voice coil, not only is the readout signal independent of the input signal driving the voice coil, but the PZT provides a substantial voltage amplitude, which provides high signal-to-noise ratio measurements. Additionally, a separate ADC (Arduino Nano microcontroller), fitted with a digital display, serves as the temperature measurement system to monitor the PMMA bulk sample temperature using a negative temperature coefficient (NTC) 10 k·ohm thermistor (Murata, NXRT15XV103FA1B010) embedded in the PMMA sample. The thermistor used a standard 10 k·ohm NTC calibration, which provides temperatures accurate to ±1.7 °C with a precision of ±0.2 °C. The temperature sensor ADC is connected to the PC for power only, as the temperatures were manually recorded when each frequency spectrum was acquired. The depth of the probe tip was set using the same gauge blocks and gauge plates to ensure a uniform probe depth of 5 mm was achieved from sample to sample.

For the experimental procedure, the PMMA samples (4.00 g total) were all prepared in the same way by using a precision electronic scale (Weigh Gram, WG-220) to measure a 50:50 by weight mixture of the methyl methacrylate monomer (MMA) (Lang Dental Mfg. Co., Jet Liquid Ref 1404); the PMAA powder (Bosworth, 166264W Duz-All White Powder) was thoroughly mixed together in a disposable aluminum weigh boat using a disposable plastic spatula for a period of 45 s. The MMA monomer liquid was dispensed with a disposable LDPE pipette directly onto the weigh boat sitting on the precision scale, and the powder PMMA was dispensed using a stainless-steel spatula. The total mass of the PMMA was measured to a precision of 0.01 g. This blended PMMA mixture was then poured into the HDPE sample container, fitted with an integral snap-top lid that had been predrilled with a 5 mm hole for probe access. The sample container was then placed on the gauge blocks on top of a tacky 1 mm thick silicone adhesive pad to ensure that the sample container did not rattle, which would contribute to noise in the signal. A series of 22 separate tests were conducted to measure the resonance spectra of the sensor probe interacting with the sample of PMMA. The sensor probe was attached to a 15 mm cross-section aluminum T slot channel rail system to provide a frame for holding the sensor probe tip above the sample in a consistent manner. The sample of PMMA was placed on a stack of gauge blocks resting on the frame to maintain a fixed distance of 5 mm from the probe tip beneath the surface of the PMMA mixture.

Once a sample of PMMA was prepared and mounted into the setup for a test, a series of acoustic excitation sweeps were taken at approximately 1 min intervals, and the raw time series data were collected and processed by software (REW) using an FFT with 8192 sample points using a Blackman–Harris window filter function to provide spectra of the signals from the PZT transducer. For each PMMA sample that was tested, typically, a series of 25 to 30 frequency spectra were obtained to ensure that the sample had fully cured and reached a steady state. Another PMMA sample was then prepared in the same way, and the test was repeated. This was carried out for 22 separate tests which required multiple days to conduct. Once all the data were acquired for this experiment, the data were checked for consistency and 3 tests were thrown out due to noise or anomalous behavior caused by either poor PMMA mix uniformity, or uneven PMMA distribution inside the sample vial. The 19 data sets that remained provide a statistically useful population to examine trends and standard deviations.

## 3. Results

**Figure 4:**
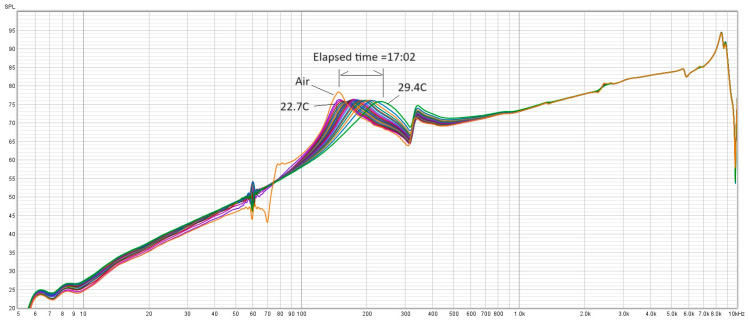
Typical full series of spectra for run 19. The free air spectrum is the yellow curve with the sharp notch at 70 Hz. The temperature starts at 22.7 °C and rises to 29.4 °C in a span of 17 min.

**Figure 5:**
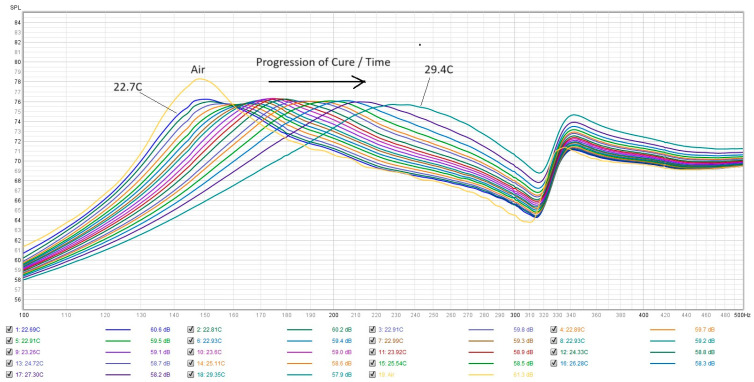
Closeup of spectra from test run 19 going through the curing stage. The corresponding temperatures for each curve are shown in the legend. The elapsed time is 17:02.

Figure 4 shows a typical frequency spectrum produced by the PZT transducer signal for PMMA undergoing the cure process. The PZT transducer has a 15 nF capacitance and the front end preamplifier for the ADC has an input impedance of 1.5 M·ohm, which gives a −3 dB high-pass first-order RC filter cutoff of about 63 Hz. Thus, for the purposes of this experiment, the measured frequency response above 100 Hz is not affected by the limited lower bandwidth of the PZT microphone. We can see that the spectrum has a rising baseline and some characteristic peaks and dips. These features are the convolution of all the acoustic–mechanical responses of the system (including the sensor, the frame, and the damping from the PMMA sample). We can observe the free air resonance frequency peak near 170 Hz. We can also see the initial resonance of freshly mixed PMMA at about the same frequency. This is to be expected as the viscosity is low. As time goes by and the PMMA starts curing, we can see that the frequency shifts to the right (blue shift) and the peak broadens (decreasing quality factor Q). If we look at a closeup of the frequency shift from the same data set, shown here in Figure 5, we can see that the frequency shift is gradual and smooth. The change in resonant frequency from 170 Hz to 320 Hz suggests that the spring constant of the DHO system must be increasing because of a reactive force onto the probe tip by the PMMA sample. This may be the result of the non-linear reactive shear stress from the increased shear thickening in the PMMA as it cures [29]. If we look at Equation (4), the resonant frequency can only be a function of the system mass and the spring constant. Since the mass has not changed, the only thing that can make the frequency increase is an increase in the effective spring constant K_TOT_.

The individual test spectra were initially processed using an in-house Python script to extract the frequency of the resonant peak. However, due to the change in the quality factor of the spectra peaks due to broadening, we found that the automated peak frequency extraction algorithm that we developed was not reliable. Consequently, the peak frequencies were manually extracted using the crosshair readout feature in the REW software. These peak frequencies were then recorded along with the corresponding measured temperature of the PMMA. The variation in the resonance frequency peaks was then fitted using least squares fit to determine the rate of change in the resonance frequency (Hz·s^−1^) over an interval up to 400 s after the start of the curing process. A 400 s duration was used to limit the extent of linear fit as it started to curve slightly upwards after that. As a result of the 400 s limit, approximately only 15 data points of the typical 30 points from each test run were used to derive the linear slope.

**Figure 6:**
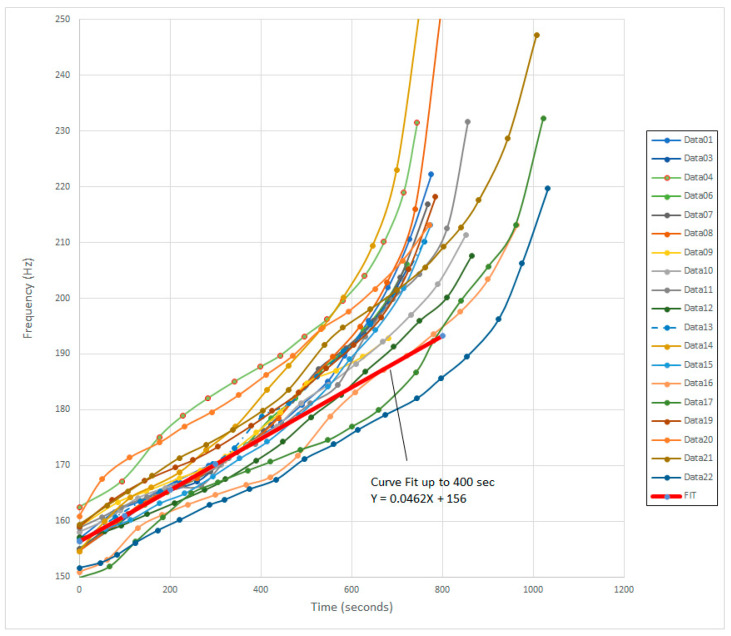
Plot of the frequency shift as a function of elapsed time (cure progress). Superposition of the data from all 19 tests is shown, plus the global average of the least squares fits for the frequency shift as a function of time for the first 400 s.

Figure 6 shows the superposition of all 19 test runs onto a single plot, and the average of all 19 least squares fitted data is then shown as a single red bold line. The data presented here represent approximately 285 data points, and thus provide a good sample to extract standard deviations. Table 1 shows the individual rate of frequency change for each test run and the ones that were anomalous and not considered for the calculation of the global average slope, as indicated by ‘n/a’. The corresponding global average slope derived from these data is 0.0464 Hz·s^−1^ ± 0.00624 Hz·s^−1^.

## 4. Discussion

As can be seen in Figure 6, when the PMMA leaves the dough state and enters the rapid setting state, the slope increases dramatically and can be used as an indicator that the cure has almost reached completion. This result can be used as a parameter in an automatic cure progress sensor to assist in the determination of the state of cure by using it to divide into the current measured frequency to compare to the initial free air resonance frequency to extract the working time still left.

**Figure 7:**
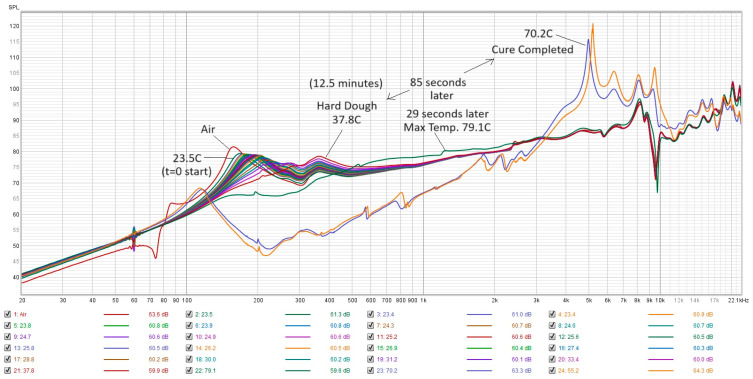
Typical collection of resonance spectra for one test run showing data all the way to fully cured PMMA. Here, the uncured PMMA (temp < 37.8 °C) versus the cured PMMA (temp = 70.2 °C) can be seen by the generation of a high-Q resonance peak near 5 kHz and large reduction in 200 Hz dough state peaks.

**Figure 8:**
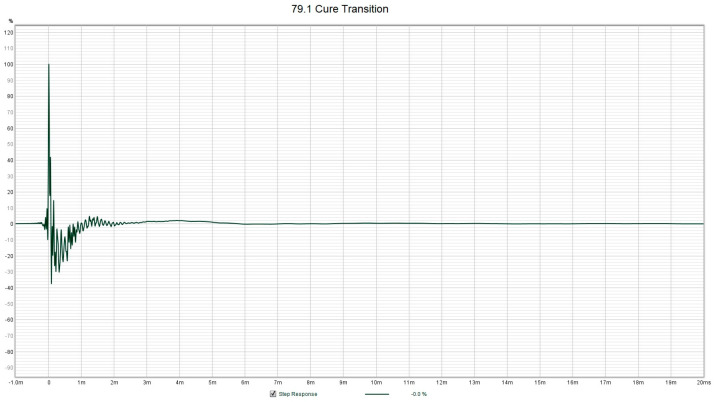
Measured step response for PMMA dough reaching a maximum temperature of 79.1 °C (setting phase) from the data shown in Figure 7. The response of soft dough is similar.

If we allow the PMMA to continue through the final setting stage to the completion of cure, the PMMA changes in hardness over a matter of tens of seconds. The hard PMMA provides an immediate high stiffness spring equivalent feedback to the sensor by showing up as the generation of a high-Q resonance peak near 5 kHz. If we look at the progression of the spectra from start to finish shown in Figure 7, we can see that the last two spectra show sharp peaks near 5 kHz at a temperature of around 70 °C. The PMMA went from a hard dough to solid PMMA in only 85 s. However, the PMMA went from hard dough at 37.8 °C to a max temperature of 79.1 °C in only 29 s, indicating a tremendous amount of heat released. It is interesting to note that the final cure state of the PMMA was not reached at the maximum temperature of 79.1 °C, but several minutes later after it has cooled to 70 °C. This is consistent with the findings in [7], where Funk et al. found that the final cure state occurred after an inflection point in the temperature, which can also be seen as a change in the sign of the first derivative of the temperature with respect to time. Once the final cure state is achieved, the PMAA is extremely hard in nature and resonates with a distinct high-Q resonance ‘note’. Figure 8 shows the step response function derived from the spectra for PMMA at a maximum temperature of 79.1 °C, which still closely resembles the step response of the cooler PMMA. The step response from an FFT measurement is the equivalent to a ring-down trace on an oscilloscope for data collected in real time, and the ring-down for Figure 8 shows a highly damped system as the energy is dissipated immediately within 1 millisecond. This is interesting because maximum temperature is used as an indicator that PMMA setting time has been reached, as per ISO 5833 [1]. But, in reality, it took about another minute for the PMMA to truly solidify into a hard plastic. This delay can vary depending on the local conditions of where the PMMA is applied (humidity, temperature, contact with bone or implants, etc.) [7].

**Figure 9:**
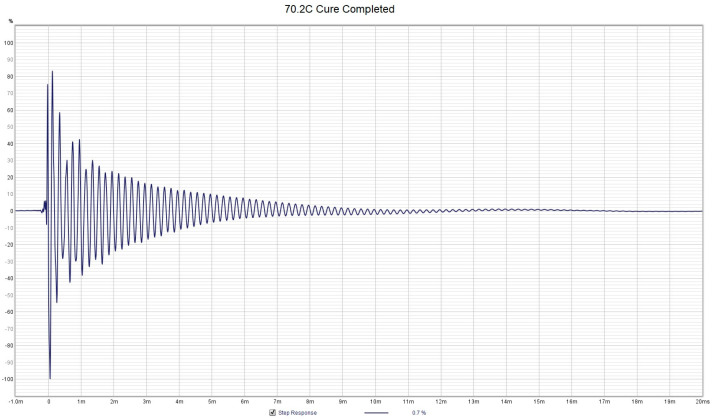
Measured step response for PMMA reaching the completion of the cure stage with a temperature of 70.2 °C from the data shown in Figure 7. The high-Q ring-down behavior at 5 kHz can clearly be observed.

However, once the PMMA has cured to a rigid plastic, the step response shows a sharp impulse followed by ring-down (under-damped harmonic oscillator) behavior with an oscillation frequency of about 5 kHz, as shown in Figure 9, which was taken 66 s after the data shown in Figure 8. The use of ring-down behavior has been used by others as an indicator of the Q of the resonance (through fitting to a model of the system as a DHO), which can be tied to the viscosity of the fluid [24]. However, for highly viscous fluids like PMMA, the ring-down may not provide a linear and well-behaved measurement property, as can be seen in the difference between Figure 8 and Figure 9, which occurred in about 2 min towards the end of the cure cycle. This sensor can thus also provide unmistakable evidence of when the PMMA has truly and fully cured, which is often another pressing question that is asked by the practitioner after the PMMA has been applied to bone and implant joints. This sensor probe tip can simply be pointed to touch the exposed grout mantle, and the ring-down step response is a clear indication that the cure phase is complete. Note that once the PMMA has hardened, neither the ring-down frequency nor the decay rate changes significantly. Table 2 summarizes the various techniques for the determination of the cure progress of PMMA and compares their accuracy, complexity, applicability in a surgical environment, and their main limitations and advantages. Starting from the left column, we have the bulk temperature of the PMMA as per the ISO 5833 standard [30], followed by manual palpation [1]; temperature derivative [7]; ultrasonic wave methods, e.g., [12]; vibrating cantilevers, which, although cannot be used with PMMA, have been included for completeness, e.g., [20]; and the axial vibrating probe of the current paper. Of these methods, only the temperature derivative method [7] and the present method using an axial vibrating probe can be used in situ or in vivo and also provide high accuracy. The main problem with the temperature and manual palpation method is that the in situ sample is affected by the moisture and temperature effects, which are different to the reference sample. The ultrasonic wave-based methods [12] require a separate sealed cell with the sample, and they also suffer from the same effects of not being representative of the in situ sample. Here, accuracy is defined as the ability to discern when a sample or its pair has reached the setting phase unambiguously.

## 5. Conclusions

We present the development of a new type of bulk-scale axially vibrating sensor to monitor the in vitro state of cure in PMMA cement. The new sensor probe uses a one-dimensional axially vibrating probe to act as a simple harmonic oscillator to sense viscosity changes. The sensor was then used to measure the acoustic spectra for 19 identical PMMA mixtures undergoing the cure process. An analysis of the data revealed positive (blue) shifts in the sensor resonance frequency with increasing cure time. This frequency shift rate can be used as a parameter to determine the relative changes in viscosity and can also be used to unambiguously determine if the PMMA sample has reached the final cure state by observing clear ring-down behavior near 5 kHz. The limitations of this new technique basically revolve around the inability to directly measure absolute viscosity without specific calibration processes and procedures for the specific electro-mechanical mass–spring–balance of the system. This technology also requires that the probe tip be manually placed or affixed to the PMMA grout under testing. However, for the purposes of use as an interoperative instrument in orthopedic surgery, where the immediate need is to discern when a PMMA mixture has elapsed beyond its nominal soft cure state and is about to undergo final cure and hardening, these limitations perhaps are not major detractions for use in the surgical setting of orthopedic bone implant surgery.

We believe that this theory can be used to develop instruments for use during surgery that will provide the surgeon with the optimal point of PMMA cementing. This ability would enhance the probability of secure fixation and potentially decrease the risk of revision surgery due to mechanical loosening. We also believe that this technique has potential uses both with PMMA during other surgeries, as well as with other processes that require a precise knowledge of fluid viscosity.

## 6. Patents

The technology described in the paper has been submitted for a patent application: USPTO Appl. No. 18/538527 filed on 13 December 2023.

## Figures and Tables

**Table 1:** Measured frequency shift rates from data linearly fitted to the first 400 s.

Test No.	Hz·s^−1^
1	0.041735
2	n/a
3	0.050174
4	0.051812
5	n/a
6	0.047088
7	0.046214
8	0.047054
9	0.039396
10	0.042141
11	0.037076
12	0.043311
13	0.045814
14	0.062565
15	0.042529
16	0.041398
17	0.057442
18	n/a
19	0.042302
20	0.048852
21	0.049051
22	0.042157
**Average**	**0.0462**
**Std. Dev.**	**0.00624**

**Table 2:** Comparison of different methods for the determination of cure progress in PMMA.

	Temperature (ISO 5833) [30]	Manual Palpation [1]	Temperature Derivative [7]	Ultrasonic Wave Propagation [12]	Vibrating Microcantilevers [20]	Axial Vibration Probe (This Paper)
Applicable to PMMA?	**Yes**	**Yes**	**Yes**	**Yes**	No	**Yes**
Accuracy *	Low	Low	**High**	Low	N/A	**High**
Complexity	**Low**	**Low**	Moderate	High	Moderate	Moderate
Practical in Surgical Environment	**Yes**	**Yes**	**Yes**	No	No	**Yes**
Use In-Situ or En-Vivo	No	No	**Yes**	No	No	**Yes**
Main Limitation	Local humidity and temperature en-vivo dramatically changes cure time	Local humidity and temperature en-vivo dramatically changes cure time	Requires probe to be inserted into sample	Requires closed sample cell with opposed acoustic faces	Cannot be used for high viscosity dough-like compounds	Requires calibration of frequency shift slope
Main Advantage	Baseline Standard	Current method used in surgical environment due to simplicity	No calibration needed, can be used in-situ or en-vivo	High Accuracy	Compact	Provides quantitative measure of cure progress

* Defined as ability to discern when in-situ or en-vivo sample pair has reached the setting phase unambiguously.

## Data Availability

The raw data supporting the conclusions of this article will be made available by the corresponding author upon request.
